# Relationship between the location of ligamentum flavum hypertrophy and its stress in finite element analysis

**DOI:** 10.1111/os.12675

**Published:** 2020-06-03

**Authors:** Yong‐xing Peng, Zhen‐yu Zheng, Wei‐guo Wang, MD, Lin Liu, Feng Chen, MD, Hong‐tao Xu, MD, Zhong‐min Zhang

**Affiliations:** ^1^ Department of Orthopaedics The Third Affiliated Hospital, Southern Medical University Guangzhou China; ^2^ Department of Orthopaedics Yingtan People’s Hospital Yingtan China

**Keywords:** Finite element analysis, Ligamentum flavum, Stress, Mechanical

## Abstract

**Objective:**

To quantitatively describe the stress of the ligamentum flavum (LF) using the finite element method and to compare the stress at different parts of the healthy LF.

**Methods:**

Based on the high resolution computed tomography imaging data of a healthy 22‐year‐old man, three‐dimensional nonlinear L_4–5_ lumbar finite element model (FEM) representing intact condition was developed. The LF, as the object of the present research, was incorporated into the spinal model in the form of solid three‐dimensional structure. The model’s validity is verified by comparing its biomechanical indices, such as range of motion and axial compression pressure displacement, with published results under specific loading conditions. To authenticate the accuracy of the solid LF, the lamina attachments, the central cross‐section, and other anatomy indicators were compared with figures in the published literature. After the average and maximum von Mises stress on the surface of LF under various working conditions were measured using ANSYS and AutoCAD software, the surface stress difference in the LF between the ventral and dorsal sides as well as the lateral and lamina parts were determined.

**Results:**

The FEM predicted a similar tendency for biomechanical indices as shown in previous studies. The lamina attachments, the central cross‐section, and the height as well as the width of the LF in the healthy FEM were in accordance with published results. In the healthy model, the average and maximum von Mises stress in the shallow layer of the LF were, respectively, 1.40, 2.28, 1.76, 1.48, 1.38 and 1.79, 2.41, 1.46, 1.42, 1.71 times that in the deep layer under a compressive preload of 500 N incorporated with flexion, extension, and lateral and rotational moments (10 Nm). The most conspicuous difference in surface stress was observed with the flexion motion, with a nearly 241% difference in the maximum stress and a 228% difference in the average stress compared to those in other states. As far as the whole dorsal side of the LF was concerned, the maximum surface stress was almost all concentrated in the dorsal neighboring facet joint portion. In addition, the maximum and average stress were, respectively, 77%, 72%, 15%, 11%, 71% and 153%, 39%, 54%, 200%, 212% higher in the lateral part than in the lamina part.

**Conclusion:**

Based on the predisposition of LF hypertrophy in the human spine and the stress distribution of this study, the positive correlation between LF hypertrophy and its stress was confirmed.

## Introduction

The ligamentum flavum (LF) is an important entity of the spinal structure. It usually starts at the antero–inferior part of the superior lamina and is attached inferiorly to the superior edge and the posterosuperior of the inferior lamina. Because of its considerable elasticity and anatomical characteristic of close proximity to the dural sac, it can not only protect the contents of the spinal canal but also play an important role in maintaining posture and body balance. In contrast, once the LF experienced pathological changes, the abovementioned functions would be seriously hampered. In addition, the ligament itself will also cause unexpected sustained compressive damage to the nervous tissue in the vertebral canal.

Contrary to the traditional belief that protrusion of the intervertebral disc is the dominating cause of compression, numerous imaging studies^1^, ^2^, ^3^ have suggested that LF contributes to approximately 50%–85% of dynamic size changes of the spinal canal. The LF occupies most of the posterior and lateral parts of the lumbar spinal canal, which is easily impacted by LF morphological and histological changes^4^. Unfortunately, these apparent pathological changes in the LF are assumed to be hypertrophy of the ligamentum flavum (HLF).

Factors such as age, sex, and disc degeneration have been reported to account for HLF. Moreover, abundant relative cell factors have been proposed to elucidate the exact mechanism through different signaling pathways. Löhr *et al*.^5^, however, showed that LF thickening was not true hypertrophy but, instead, reflected its extreme degeneration and final fibrosis. Similarly, Yoshiiwa *et al*.^6^, ^7^ demonstrated that histological alterations were homologous to tissue scarring during the post‐inflammatory repair process in other organs. It was also found that inflammatory cells were infiltrated around the degenerative LF. Therefore, the inflammatory theory for the etiology of LFH is supported by many researchers.

On this basis, Nakatani *et al*.^8^ further explained the cause of the inflammation. The specific mechanisms were confirmed by cell culture in *in vitro* and cell tension membrane experiments on LF. In the study, free yellow ligament cells were divided into tension and no tension groups. After 24 and 48 h of continuous stress, mRNA expressions of transforming growth factor‐β1, and type III and V collagen fibers of yellow ligament cells were significantly increased in the tension group. Meanwhile, the expression of the 48‐h tension group was higher than that of the 24‐h tension group. Transforming growth factor‐β1 can induce the proliferation of fibroblasts and plays a pivotal role in the formation of tissue fibrous lesions, such as cirrhosis and renal fibrosis. Consequently, scholars have reached the consensus that mechanical stress may be the original trigger for an inflammatory response and subsequent scar development.

Hitherto, many studies have been conducted to confirm this theory, using, for instance, animal models^9^, ^10^, and radiological^6^, histological^11^, ^12^, and cell mechanics analysis^8^. However, these findings are indirect and not convincing. For example, most animal models were established using quadrupedal animals, such as mice; therefore, the spinal activity patterns may be distinct to those of bipedal humans; the change in imaging indicators, such as segmental angulation and facet angle, fail to quantitatively describe stress discrepancies in the LF. Moreover, studies on the mechanisms of HLF are mostly confined to the molecular, cellular, or histological level, while studies applying the human spinal disease model in which mechanical changes in the LF can be measured are relatively rare. Finally, limited data exist to discuss stress differences between the peripheral portion of the facet joint^8^ and other areas, in addition to between superficial and deep layers^13^, thus contributing to LFH prevalently occurring in the joint capsule and on the dorsal side.

The finite element method (FEM) takes full account of the changing substantial parameters to comprehensively understand the function of each part and circumstance in spinal kinematic reactions, with the great advantage of obtaining stress–strain distribution in various tissues that are difficult measured using traditional methods. Hence, we can simulate the working conditions of diverse spinal motions, in which the stress of LF can be numerically quantitated to assess LF biomechanics under various physiological movements. After the stress difference in each part of the LF is calculated, and then combining with the LFH predisposing site, we can determine the relationship between the stress and LF hypertrophy.

Based on the aforementioned insufficient solid evidence and useful tools, the aims of current study were threefold: (i) to establish and verify a 3D nonlinear FEM of the lumbar (L_4–5_), (ii) to compare the surface stress between the shallow layer and the deep layer in a healthy functional spinal unit (FSU), and (iii) to identify the dissimilarity of dorsal surface stress between the lateral part and the lamina part in the healthy model. We speculate that the LF sites with higher stress have a higher probability of HLF.

## Materials and Methods

### *Establishment of the Healthy Finite Element Model*

Based on the principles proposed by Yoganandan^14^, the present research, which was approved by the ethics committee of the hospital, was focused on a healthy 22‐year‐old man who provided written informed consent. The geometric shape of the L_4–5_ FSU was generated from his high‐resolution CT (Siemens, Munich, Germany) imaging data that showed no evidence of any pathological lesions. We imported the CT imaging data into Mimics software (v10.01 Materialize, Leuven, Belgium) to build a 3D vertebral model of L_4–5_ FSU. Because the surface of the 3D vertebral model was very rough at this time, the image was then smoothed using Geomagic studio software (v2013 Raindrop company, Marble Hill, USA), which was beneficial to the later mesh division and could enhance the convergence of the calculation. Following that, the 3D vertebral model was transformed into a solid model using SolidWorks software (v2012 Dassault Systèmes SA, Massachusetts, USA) in which vertebral bodies were divided into cancellous bone, cortical bone, and posterior bony elements including the facet articulations. Meanwhile, the annulus, the nucleus, the cartilaginous, bony endplates, and six major ligaments (excluding the LF) were added based on the work of Ellingson *et al*.^15^. As for the LF, it was simulated based on preoperative MRI (Philips, Amsterdam, the Netherlands). Finally, after the solid model was meshed and assigned properties such as Young’s modulus and Poisson’s ratio, the mechanical analysis of the model was calculated in ANSYS software (v2012 Ansys, Pennsylvania, USA), using unconstrained moment loads.

**Figure 1:**
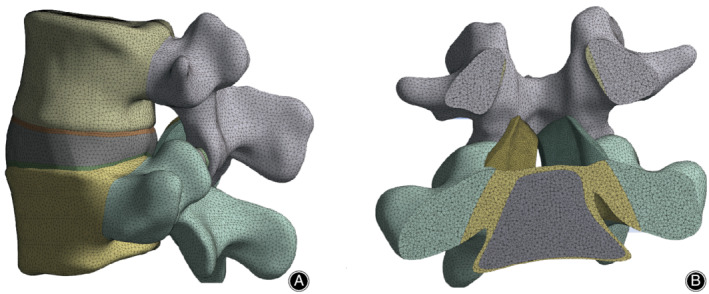
Element meshes of the L_4,5_ motion segment with a healthy disc (A) and section view of the ligamentum flavum (B).

The components of the healthy model setting were based on the model of Ellingson *et al*.^15^, with the following differences: (i) all solid constructions were modeled by four‐node tetrahedral solid elements; (ii) all ligaments merged into the model, excluding LF, were represented as two‐node spring elements with nonlinear isotropic hyperelastic material properties; (iii) the LF was also defined as having hyperelastic material properties and four‐node tetrahedral solid elements to match those of the whole model; and (iv) collagen fibers were not defined in the intervertebral disc. The complete intact FEM is shown in Fig. 1. Figure 1A,B displays lateral and section views of the FEM, respectively. The parameters and references are presented in Table 1 16, 17, 18, 19, 20.

**Table 1:** Material properties used in the finite element healthy model

Materials	Young’s modulus (MPa)	Poisson’s ratio	Cross‐section (mm^2^)	References
Vertebra				
Cortical bone	12,000	0.3	—	Cassidy *et al*.^16^
Cancellous bone	100	0.2	—	Goulet *et al*.^17^
Posterior elements	3500	0.25	—	Shirazi‐Adl *et al*.^18^
Disc				
Nucleus pulposus	1	0.499	—	Pitzen *et al*.^19^
Annulus fibrosis	4.2	0.45	—	Pitzen *et al*.^19^
Endplate	24	0.4	—	Shirazi‐Adl *et al*.^18^
Ligament				Goel *et al*.^20^
Anterior longitudinal ligament	7. 8 (ε < 12. 0%), 20. 0 (ε > 12. 0%)	—	75.9	
Posterior longitudinal ligament	10. 0 (ε < 11. 0%), 20. 0 (ε > 11. 0%)	—	51. 8	
Transverse ligament	10. 0(ε < 18. 0%), 58. 7 (ε > 18. 0%)	—	2. 0	
Capsular ligament	7. 5(ε < 25. 0%), 32. 9(ε > 25. 0%)	—	102.5	
Interspinous ligament	10. 0(ε < 14. 0%), 11. 6 (ε > 14. 0%)	—	36.3	
Ligamentum flavum	15. 0(ε < 6. 2%), 19. 5 (ε > 6. 2%)	—	78.7	
Supraspinous ligament	8. 0(ε < 20. 0%), 15. 0(ε > 20. 0%)	—	75. 7	

### *Boundary and Loading Conditions*

Loading of the FEM mimicked the operation process of the experimental protocol under a compressive preload of 500 N with bending moments (10 Nm) in three principal directions. Loads were applied at the center of the upper surface of the L_4_ vertebral body provided that the inferior endplate of the L_5_ vertebra was strictly fixed^21^, ^22^, ^23^, ^24^, ^25^, ^26^.

### *Validation*

To test the validity of different FEM, the anatomic morphology was compared to those reported in the published literature or their predictions to experimental data gained *in vitro* under the same loading conditions.

### *Biomechanical Analysis of Ligamentum Flavum*

#### *Maximum von Mises Stress*

Von Mises stress, which is actually an equivalent stress, is usually used to represent the stress distribution in a model and can clearly demonstrate the result of stress change in the whole model. Just as its name implies, maximum von Mises stress refers to the maximum von Mises stress of a certain part, in which the most vulnerable area in the model recognized by biomechanics specialists. Typically, maximum von Mises stress on the surface of LF can be displayed directly in the ANSYS software.

#### *Average von Mises Stress*

**Figure 2:**
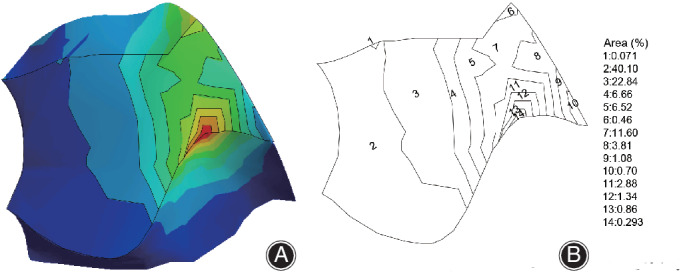
The method for calculating average stress using AutoCAD software. (A) After we imported the surface stress nephogram into AutoCAD software, the contour lines of each color in the surface stress nephogram were drawn using AutoCAD software. (B) Following the total area and the area represented by each color being measured, the percentage of each color in the total area was calculated.

Generally speaking, average von Mises stress indicates the total stress of a certain part of a model. It is an indicator of whether a part of the model is easy to damage compared with other components. The measuring method of average surface stress in this study is shown in Fig. 2. The outcome of multiplying the median stress of different chromatograms by the percentage value was the respective stress. The average surface stress was defined as the total numerical value of the respective stress.

#### *Research Indicators in This Study*

The testing in the present study includes: (i) calculating the average and maximum von Mises stress on the surface of LF under various working conditions; (ii) comparing surface stress difference in LF between the ventral and dorsal sides in the healthy model; and (iii) determining whether there is LF surface stress diversion between the lateral and lamina parts in the intact model.

## Results

### *Validation Results*

#### *Ligamentum Flavum*

The superior and inferior lamina attachments of the LF in the healthy FEM were located in the inferior and superior borders, respectively, which was in accordance with the type I classification system described by Chau *et al*.^27^. In all four types, type I LF attachment accounted for 26%. The central cross‐section (excluding the lateral part) of the LF on the right and left sides measured in SolidWorks software was 38.64 and 39.27 mm^2^, respectively. The indicator was perfectly close to the outcome reported by Kim *et al*.^28^, ^29^. The LF height and width illustrated by Akhgar *et al*.^30^ were measured as 14.65 and 22.25 mm, respectively, which were within one standard deviation of the *in vitro* outcomes at the L_4–5_ level.

#### *Healthy Finite Element Model*

**Figure 3:**
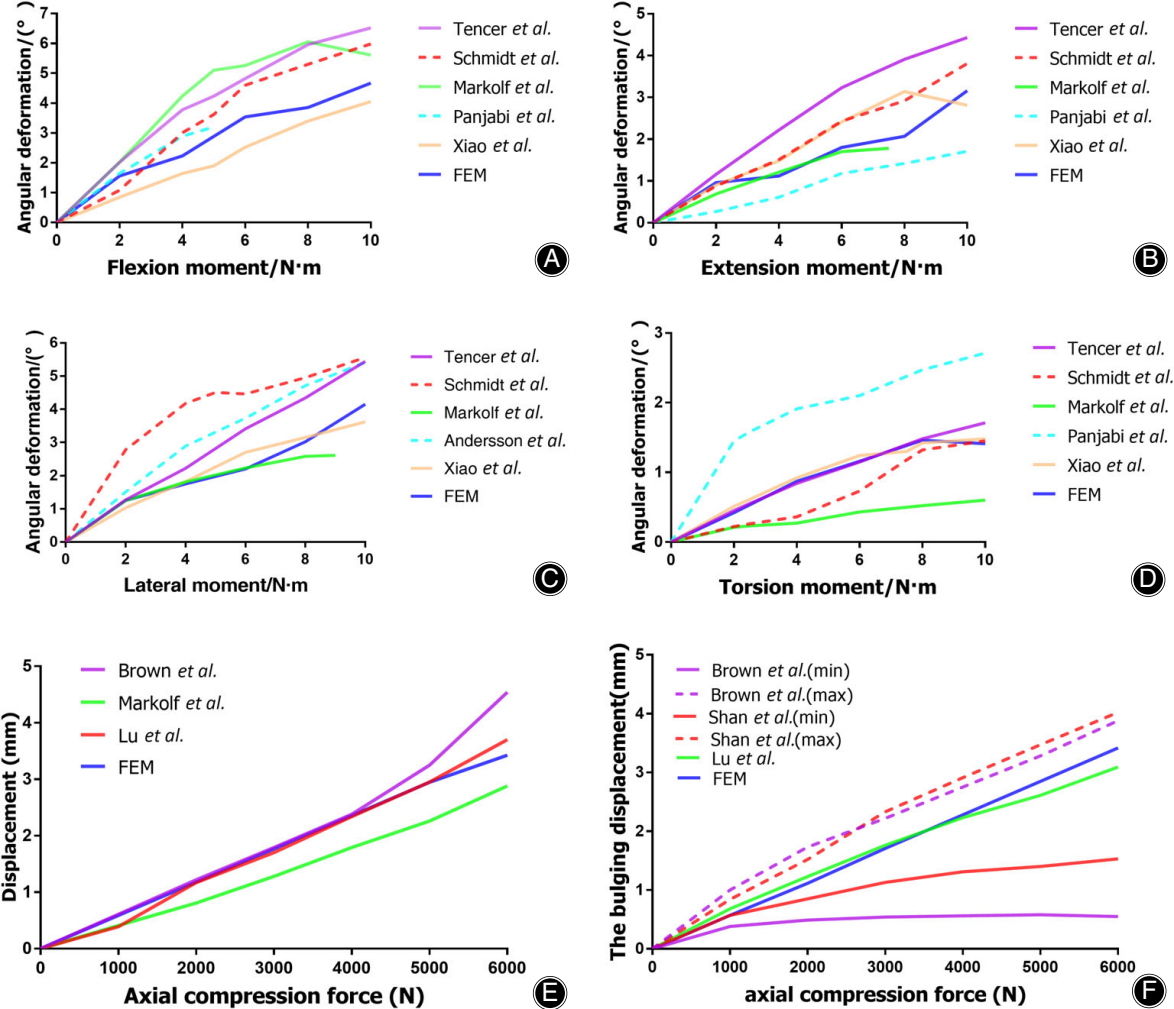
Comparisons between our results and the results from published data in a healthy model. Range of motion (ROM) under flexion moment (A), extension moment (B), lateral moment (C), torsion moment (D), axial compression pressure‐displacement curve of the motion segment (E), and axial pressure‐bulging displacement curve of the posterolateral disc (F).

The comparisons are shown in Fig. 3. The angular deformations in three fundamental directions (Fig. 3A‐D) were within the range of the curves obtained in other published studies^21^, ^22^, ^23^, ^24^, ^25^, ^26^ and the torsion moment significantly corresponded with the relevant curve. The axial and posterior annulus fibrosus bulge displacement increased linearly with increasing load. The axial displacement (Fig. 3E) was consistent with that reported in previous studies^31^, ^32^, ^33^. The curves of the posterior disc bulge displacement (Fig. 3F) were between those demonstrated by Brown^32^ and Shah^34^. In addition, they were extremely close to the curves demonstrated by Lu *et al*.^31^.

### *Ligamentum Flavum Biomechanical Study in Intact Model*

#### *Maximum and Average von Mises Stress*

**Figure 4:**
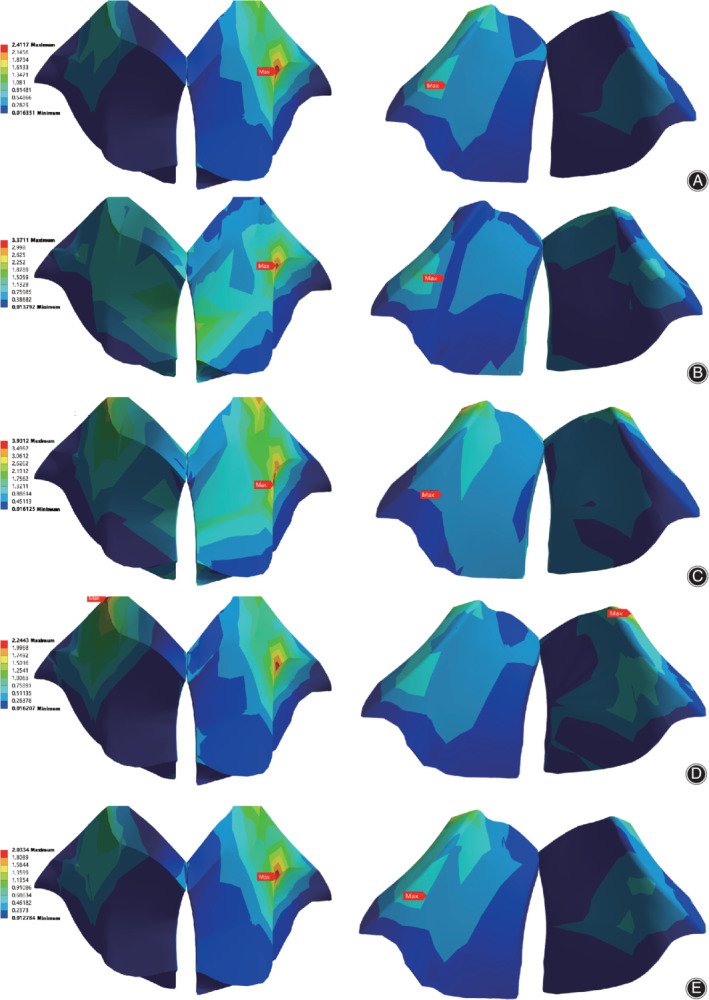
The surface stress nephogram of the ligamentum flavum dorsal (left) and ventral (right) side in the intact model. Under a 500 N compressive load (A) and preload plus bending moments of 10 Nm in flexion (B), extension (C), lateral bending (D), and axial rotation (E).

The surface stress nephograms of the healthy model under five different working conditions are shown in Fig. 4. Figure 4A–E represent the surface stress nephogram of the LF when the spinal segment was subjected to passive vertical compression, flexion, extension, lateral bending, and axial rotational motion, respectively. The left and right sides of Fig. 4A–E show the stress nephogram of the dorsal and ventral sides of the LF in different motions. As can be clearly seen from Fig. 4, no matter what type of motion, the maximum surface stress of the spine was almost all concentrated in the dorsal neighboring facet joint portion. Next, based on the chromatograms, we determined the average von Mises stress using AutoCAD software (v2019 Autodesk, California, USA) according to the calculation method noted above. Detailed values of the maximum and average von Mises stresses are shown in Table 2.

**Table 2:** The specific value of the maximum and average von Mises surface stress of ligamentum flavum (MPa)

Finite element model	Preload	Flexion	Extension	Bending	Rotation
Maximum stress					
Dorsal side	2.41	3.37	3.93	2.24	2.03
Ventral side	1.35	1.40	2.70	1.58	1.19
Dorsal lateral part	2.41	3.37	3.93	2.24	2.03
Dorsal lamina part	1.36	1.96	3.42	2.01	1.19
Average stress					
Dorsal side	0.39	0.89	1.04	0.43	0.33
Ventral side	0.28	0.39	0.59	0.29	0.24
Dorsal lateral part	0.81	1.15	1.48	1.02	0.78
Dorsal lamina part	0.32	0.83	0.96	0.34	0.25

#### *Surface Stress Difference in Ligamentum Flavum between the Ventral and Dorsal Sides*

**Figure 5:**
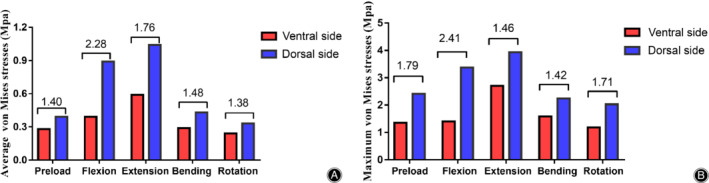
Average (A) and maximum (B) surface von Mises stress in ventral and dorsal side of the ligamentum flavum under five kinds of load conditions. The numerical values in the figure are multiples of the pressure value of the dorsal side compared with that of the ventral side.

The results are presented in Fig. 5. Figure 5A and B demonstrate the maximum and average surface stress in the superficial (red column) and deep (blue column) layer of the LF, respectively. The numerical value in the figure makes it easy to detect the surface stress difference between them. As can be seen from Fig. 5, regardless of the maximum or the average value, the surface stress on the dorsal side was noticeably greater than that on the ventral side. The maximum dorsal surface stress was almost concentrated at the junction of the LF and the facet joint, whereas the maximum ventral surface stress was at the junction of the ligament and the upper lamina. Furthermore, the surface stress was at its maximum for the extension motion relative to other motions. From the figure we can also see that the most conspicuous difference in surface stress was observed for the flexion motion, with nearly a 241% difference in the maximum stress and 228% difference in the average stress compared to those in other states.

#### *Surface Stress Diversion in Ligamentum Flavum between the Lateral and Lamina Parts*

**Figure 6:**
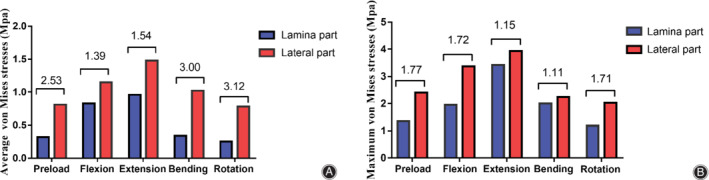
Average (A) and maximum (B) surface von Mises stress in dorsal lamina and lateral part of the ligamentum flavum under five kinds of load conditions. The numerical values in the figure are multiples of the pressure value of the dorsal lateral part compared with that of the lamina part.

As shown in Fig. 6, the surface stress difference in the lateral and lamina parts of the LF dorsal side could also be easily observed. Figure 6A and B, respectively, display the maximum and average surface stress in the lamina and lateral parts of the LF. The numerical value in the figure is a multiple of the stress value of the dorsal lateral part compared with that of the lamina part. Judging from Fig. 6, the maximum surface stress of the lateral part was equal to that of the entire dorsal side. The difference in lateral and lamina parts was also obvious, except that the tendency of maximum surface stress was relatively unremarkable.

## Discussion

An appropriately developed and validated FEM can be a useful means to noninvasively evaluate multifarious motion states of the human body. In the present study, the dynamic changes of the LF in an intact spinal model were assessed by means of FEM. Through mechanical analysis, we found that the stress of the LF was closely related to its hypertrophy.

### *Establishment of the Model and Verification of its Validity*

Based on the original image data of the patient, we used the traditional methods to build the FEM and the setting of its components, referring to the literature of others. To verify the validity of the model, the FEM predicted a similar tendency for biomechanical indices as shown in previous studies. To authenticate the accuracy of the research object, the lamina attachments, the central cross‐section, and other anatomy indicators of the LF in the FEM were compared with published results.

### *Surface Stress Difference in Ligamentum Flavum between the Ventral and Dorsal Sides*

At the maximum or average value of the motion of the three principal planes, the surface stress on the dorsal side was markedly greater than that on the ventral side. A related study^35^ also demonstrated this result, illustrating that the dorsal layer had obviously increased stress when compared to the dural layer at all spinal segments and for all movements, except extension. The biomechanical research was also performed by FEM, but the LF consisted of two‐layer cable elements, representing the dorsal and ventral parts of the ligament. Different from our results, the LF did not experience any stress during extension motion. These discrepancies in extension may be attributed to the model consisting of four vertebral bodies, L_3_–S_1_, meaning the longitudinal LF direction tilts forward. Under these conditions, the LF was mainly influenced by pressure stress rather than tension stress. The effect of compressive stress on the ligament was not particularly apparent because of its own structural characteristic. Furthermore, ligament properties were usually endowed with tension only in ANSYS software, thereby leading to quite divergent results. Likewise, the difference in ligament stress was confirmed by histopathology. Based on results of the Elastica van Gieson (EVG) staining section of the LF in different groups, Kosaka *et al*.^36^ observed that elastic fibers inevitably lessen with age on the superficial layer but not obviously on the deep layer. Furthermore, the dorsal layer was more easily identified by converting from elastic composition to cartilaginous or scar composition as age increased.

### *Surface Stress Diversion in Ligamentum Flavum between the Lateral and Lamina Part*

The LF surface stress of the lateral and lamina parts was subsequently measured. Surprisingly, the stress displayed higher values in the lateral part. Moreover, the maximum stress was almost concentrated in the neighboring articular capsular portion. Although there was no direct evidence to support that the LF in the facet joint was inclined to develop HLF, in practice, there evidence to indirectly support the view. Yoshida *et al*.^37^ found that massive proliferation of collagen type II in the capsular portion of the LF clings to the facet joint. Hayashi *et al*.^8^ substantiated the result in a novel animal model where the stress between L_3_ and L_4_ vertebra increased by fusing the upper and lower segments. Through EVG and toluidine blue staining analysis, the collagen, cartilage matrix augmentation, and chondrocyte‐like cell proliferation are likely to be seen in the same location at L_3_–L_4_ level. By contrast, the imaging study provided more information; imaging indicators such as facet joint osteoarthritis and facet tropism^38^, ^39^ are frequently reported to be associated with LFH development. Based on our experience, the reason for this finding might be the fact that the articular capsules are the turning point between the lamina and lateral parts; there is a tendency for stress concentration on it after load, leading to its higher stress value than that in other regions.

### *Limitations and Innovations of the Study*

To the best of our knowledge, there are two innovations in the study. First, the LF in the spinal FEM is set to solid instead of linear. In addition, after the stress nephogram of the ligamentum flavum was obtained, we used AutoCAD software to calculate the average surface stress of the LF.

Just like any FEM that attempts to simulate the diversity of the human body, the present study is also limited by only focusing on one functional spinal unit (L_4–5_), and multi‐segmental loading might discern level differences. Another intrinsic limitation is that simplifications in regard to material property and diversity in attributes between individuals may bring about different biomechanical results. In addition, mechanical loading conditions are confined to the fundamental direction of motion, which is quite different from the fact that the spine experiences various coupling loading patterns. Finally, the anatomy of the LF itself is quite distinguishing; for instance, there are several variations of the LF in the midline attachment between left and right, and lateral extent to neural foramen. Adding to the number of spine segments and increasing the sample capacity of study models in further studies may be beneficial.

### *Conclusion*

This study quantitatively described the stress of the LF using the FEM and compared the stress at different parts of the LF in a healthy model. Under the same conditions, the surface stress on the dorsal and facet joint of the LF was higher than in other parts. These stress differences are significant for understanding the mechanism of HLF. Combined with the predisposition of LF hypertrophy in the human spine and the mechanical distribution of this study, the results confirmed that excessive mechanical stress usually results in HLF.
